# Association between alpha-1-acid glycoprotein and infertility in the NHANES 2015–2020

**DOI:** 10.1097/MD.0000000000046749

**Published:** 2025-12-26

**Authors:** Jia Lei, Xiaohua Zhong, Xiaohong Lin, Meng Zhang

**Affiliations:** a Department of Dermatology, People’s Hospital of Changshou Chongqing, Chongqing, China.

**Keywords:** alpha-1-acid glycoprotein, infertility, inflammation, NHANES

## Abstract

Infertility is a prevalent reproductive disorder, with local and systemic inflammatory responses potentially playing a pivotal role in its etiology. Alpha-1-acid glycoprotein (AGP), an acute-phase glycoprotein, has been implicated in inflammatory processes. This study set out to find out the relationship between AGP levels and infertility among American women. A cross-sectional survey was used for analyzing the National Health and Nutrition Examination Survey data from 2015 to 2020. For study of the relationship between AGP and infertility, multiple logistic regression models were utilized. Smoothed curve fitting and threshold effects were implemented to describe the nonlinear relationship between the two. The study consisted of 2229 female volunteers between the years of 18 and 45, with 262 cases of infertility. Both Model 1 (OR = 2.97, 95% CI: 1.76, 5.00), which was not adjusted for covariates, and Model 2 (OR = 2.60, 95% CI: 1.50, 4.49), which was corrected for age and race, revealed a significant positive association between AGP and infertility. However, after controlling for all variables, this positive relationship was insignificant in model 3 (OR = 1.44, 95% CI: 0.68, 3.08). The link between AGP and infertility failed to significantly depend on the impact of each subgroup (*P* for all interaction tests > 0.05). A positive nonlinear link between AGP and infertility was identified utilizing smooth curve fitting. Furthermore, threshold effect analysis exhibited an inverted U-shaped connection with an inflection point of 0.76 g/L between AGP and infertility in women elderly 35 and above. According to our research, AGP and infertility are associated positively. It might inspire novel approaches to clinical care, but additional investigation is required to comprehend the underlying mechanisms.

## 1. Introduction

Infertility, defined as the inability to achieve a clinical pregnancy after 12 months of unprotected intercourse, is a prevalent reproductive disorder.^[1]^ Globally, it affects an estimated 186 million individuals, with 8% to 12% of couples of reproductive age experiencing infertility, and this prevalence is escalating annually.^[2,3]^ The etiology of female infertility is multifactorial, encompassing ovulatory dysfunction, tubal disease, genetic predispositions, infections, and modifiable lifestyle and environmental factors such as smoking, excessive alcohol intake, and obesity.^[4]^ Notably, polycystic ovary syndrome (PCOS) and endometriosis are identified as the most prevalent conditions contributing to infertility,^[5]^ with an excessive inflammatory state being a key pathophysiological factor.^[6,7]^

Alpha-1-acid glycoprotein (AGP) is an acute-phase protein synthesized by the liver and peripheral tissues in response to systemic inflammation.^[8]^ Extensive research has established a robust correlation between AGP levels and the prognosis of various malignancies, including breast cancer,^[9,10]^ lung cancer,^[11]^ and melanoma,^[12]^ as well as non-neoplastic conditions such as nephropathy^[13]^ and sepsis.^[14]^ Furthermore, AGP is a key immunomodulatory glycoprotein in reproductive health. Structurally, AGP’s heavily sialylated glycans enable specific immunomodulatory functions: its terminal sialic acid residues bind to siglec receptors on endometrial macrophages, polarizing them toward an anti-inflammatory M2 phenotype under physiological conditions.^[15]^ However, in pathological states like endometriosis, AGP undergoes aberrant fucosylation, upregulating pro-inflammatory cytokines (IL-6, tumor necrosis factor α [TNF-α]) that disrupt implantation.^[16]^ Clinically, AGP levels > 0.85 g/L correlate with 3.0-fold higher infertility risk in PCOS patients - a relationship largely mediated through insulin resistance-induced glycoprotein hypersecretion.^[17]^ Despite these condition-specific associations, population-level data examining AGP-infertility links in ethnically diverse cohorts remain scarce, highlighting the novelty of our NHANES-based investigation.

Consequently, the present study aimed to elucidate the association between α1-acid glycoprotein (AGP) and infertility by examining a population-based sample of women aged 18 to 45 years, drawn from the National Health and Nutrition Examination Survey (NHANES) spanning the years 2015 to 2020. The goal was to produce fresh insight that could guide clinical approaches for managing infertility.

## 2. Materials and methods

### 2.1. Study population

NHANES is a cross-sectional survey that has been carried out in the United States. It is an exhaustive database that contains information on questionnaires, laboratory test outcomes, dietary information, and demographics. The NCHS Ethics Review Board has approved all NHANES study methods, all survey participants have given written informed consent, and all NHANES data are available at www.cdc.gov/nchs/nhanes/.

**Figure 1.**
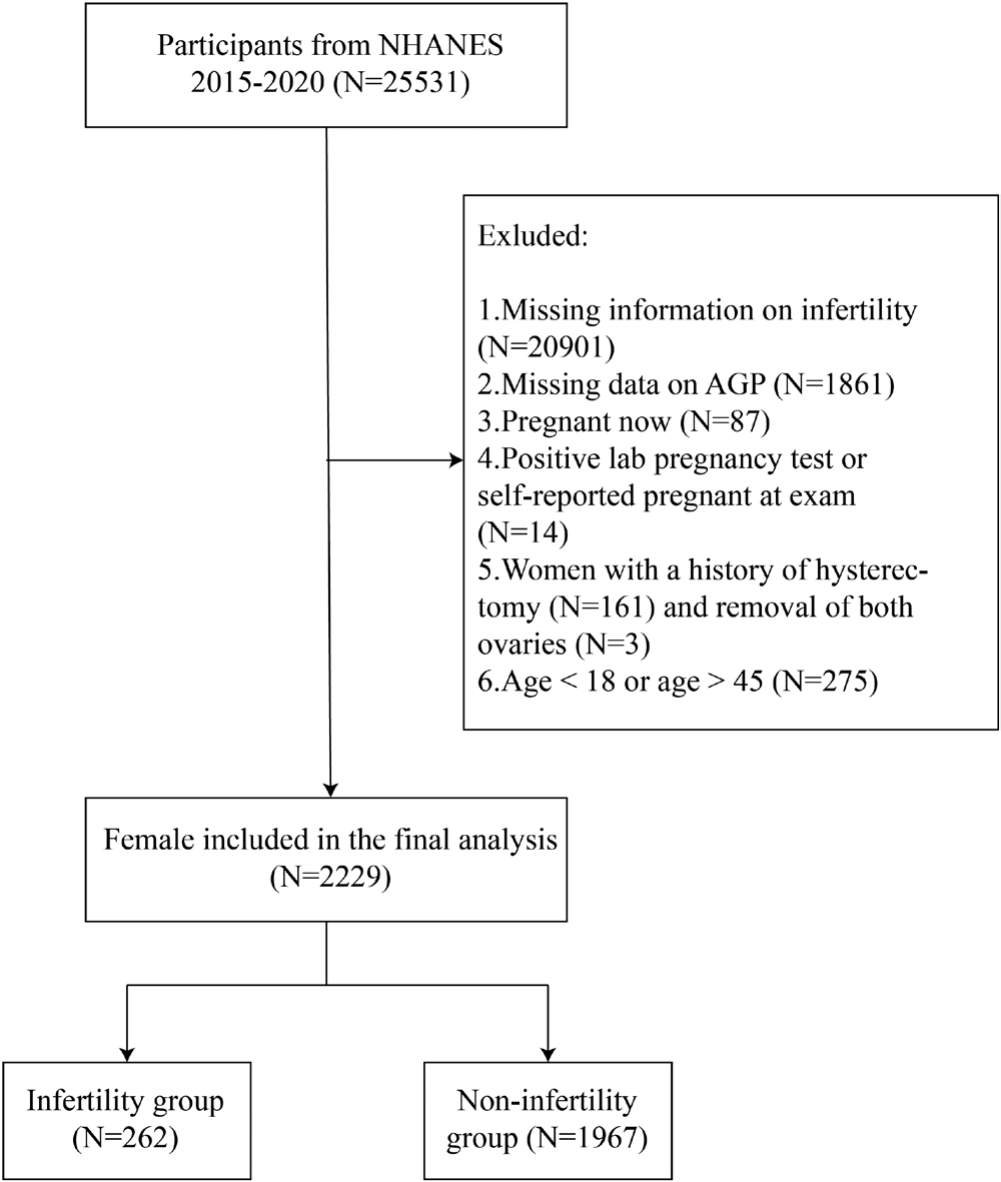
Flowchart of participant selection. AGP = alpha-1-acid glycoprotein, NHANES = National Health and Nutrition Examination Survey.

This study collected NHANES data from 2015 to 2020 and excluded 20,901 cases with missing infertility data, 1861 cases with missing AGP data, and 275 cases aged less than 18 or greater than 45. Additionally, individuals with hysterectomy (n = 161) or bilateral oophorectomy (n = 3) were excluded, as these procedures inherently cause sterility, confounding infertility unrelated to AGP. Furthermore, 87 pregnant individuals and 14 with positive lab pregnancy tests or self-reported pregnancy at the examination were excluded to avoid transient physiological changes in AGP. In the end, 2229 individuals participated in the study (Fig. 1).

### 2.2. Study variables

Infertility, which was generated from 2 questions in the Reproductive Health Questionnaire (RHQ074 and RHQ076), was the outcome variable in this study, and AGP was the exposure variable. “Have you ever attempted to become pregnant over a period of at least a year without success?” asked the question posed by Question RHQ074. “Have you ever consulted a doctor or medical provider because you were unable to become pregnant?” was the question requested by RHQ076. Infertility was indicated by a positive answer to either question.

Covariates were selected a priori based on established associations with infertility or potential confounding relationships with AGP, including: reproductive health factors: pelvic inflammatory disease history, age at menarche, menstrual cycle regularity, and female hormone use; metabolic parameters: diabetes, hypertension, and high cholesterol levels; anthropometric measures: weight, body mass index (BMI), and waist circumference; sociodemographic characteristics: age, race, educational attainment, marital status, and poverty-income ratio; and lifestyle exposures: smoking status and alcohol consumption – with diabetes, hypertension, and high cholesterol determined via self-reported questionnaire data, while all other variables were obtained through standardized NHANES examinations and laboratory measurements.

### 2.3. Statistical analysis

The statistical calculations were carried out with R software (version 4.1.3) and EmpowerStats (version 2.0) with a significance threshold of *P* < .05. The continuous variables in the participant baseline tables were presented using mean ± standard deviation, as categories of information were expressed given percentages (%). Multiple logistic regression method was adopted to evaluate the link between AGP and infertility, with the calculation of odds ratios (OR) and their respective 95% confidence intervals to determine the strength and reliability of the associations. The research encompassed 3 distinct models for analysis: the initial model, which was unadjusted; the subsequent model, which took age and gender into account; and the final model, which included adjustments regarding potential confounding variables. Additionally, threshold effects analyses, smoothed curve fitting, and subgroup analysis were carried out to evaluate the link and inflection points between AGP and infertility.

## 3. Results

### 3.1. Baseline characteristics of participants

With an average age of 31.15 ± 8.27 years, 2229 women between the ages of 18 and 45 were eventually included in this study. Of these, 262 women were identified as having infertility. The mean serum level of AGP, reported with its standard deviation, was 0.788 ± 0.238 g/L.

The baseline characteristics of the subjects, categorized by infertility status, are shown in Table 1. Women with infertility were older, and their body weight, BMI, waist circumference, and AGP measures were higher than those of the non-infertile group. In addition, they were more likely to be married or cohabit together, and while their percentage of poverty was lower, they were more likely to have diabetes, hypertension, hypercholesterolemia, pelvic inflammatory disease, irregular menstruation, usage of female hormones, and a history of smoking and alcohol use.

**Table 1:** Basic characteristics of participants (N = 2229).

Characteristics	Infertility N = 262	Control N = 1967	*P* value
Age (yr)	34.89 ± 6.84	30.88 ± 7.95	<.0001
Race/ethnicity (%)			.5852
Mexican American	14.35	12.34	
Other Hispanic	6.81	8.39	
Non-Hispanic White	60.96	57.59	
Non-Hispanic Black	9.75	11.33	
Non-Hispanic Asian	4.52	5.77	
Other Races	3.61	4.59	
Education level (%)			.7794
Less than high school	9.98	9.76	
High school or GED	20.90	19.19	
Above high school	69.12	71.05	
Marital status (%)			<.0001
Married/living with partner	75.90	56.91	
Widowed/divorced/separated	12.05	7.91	
Never married	12.05	35.18	
Diabetes (%)			<.0001
Yes	7.58	2.66	
No	97.34	92.42	
Hypertension (%)			.0051
Yes	15.75	10.17	
No	84.25	89.83	
High cholesterol level (%)			<.0001
Yes	20.42	11.30	
No	79.58	88.70	
Smoked at 100 least cigarettes (%)			<.0001
Yes	41.05	29.56	
No	58.95	70.44	
Have 4/5 or more drinks every day (%)			.0349
Yes	10.05	6.47	
No	89.95	93.53	
PID (%)			<.0001
Yes	8.88	3.46	
No	91.12	96.54	
Age at menarche, years (%)			.7749
<15	87.05	86.43	
≥15	12.95	13.57	
Menstrual cycle regularity (%)			.0215
Yes	88.09	92.16	
No	11.91	7.84	
Female hormones (%)			.0004
Yes	7.95	3.45	
No	92.05	96.55	
PIR	2.97 ± 1.65	2.73 ± 1.66	.0278
BMI (kg/m^2^)	31.64 ± 8.74	28.98 ± 8.13	<.0001
Weight (kg)	84.77 ± 24.29	76.69 ± 22.62	<.0001
Waist circumference (cm)	102.39 ± 19.36	94.17 ± 18.23	<.0001
Alpha-1-acid glycoprotein (g/L)	0.85 ± 0.23	0.78 ± 0.24	<.0001

Mean ± SD for continuous variables: *P* value was calculated by weighted linear regression model.

% for categorical variables: *P* value was calculated by weighted chi-square test.

BMI = body mass index, PID = pelvic inflammatory disease, PIR = ratio of family income to poverty.

### 3.2. Association between AGP and infertility

Table 2 delineates the association between AGP levels and the risk of infertility. A 1.97-fold increased risk of infertility was associated with a 1 g/L increase in AGP in the crude Model 1 (Model 1: OR = 2.97, 95% CI: 1.76–5.00). This increase was linked to a 1.6-fold higher risk of infertility after controlling for age and ethnicity in Model 2 (Model 2: OR = 2.60, 95% CI: 1.50–4.49). In contrast, a non-significant positive association between AGP and infertility was seen in the fully adjusted Model 3, which included all confounders (Model 3: OR = 1.44, 95% CI: 0.68–3.08). In both Model 1 and Model 2, the significance of the positive relationship was maintained by stratifying AGP into quartiles. Interestingly, although the association in the fully adjusted Model 3 was generally non-significant, a substantial positive association with infertility was observed within the AGP range of 0.614–0.769 g/L (Model 3: OR = 1.84, 95% CI: 1.11–3.04).

**Table 2:** Association between alpha-1-acid glycoprotein and infertility.

	Model 1 OR (95% CI) *P* value	Model 2 OR (95% CI) *P* value	Model 3 OR (95% CI) *P* value
AGP (g/L)	2.97 (1.76, 5.00) <.0001	2.60 (1.50, 4.49) .0006	1.44 (0.68, 3.08) .3422
AGP (quartile)			
Q1 (0.262–0.613 g/L)	Reference	Reference	Reference
Q2 (0.614–0.769 g/L)	1.61 (1.07, 2.43) .0229	1.54 (1.01, 2.33) .0439	1.84 (1.11, 3.04) .0182
Q3 (0.77–0.945 g/L)	1.85 (1.24, 2.77) .0028	1.66 (1.09, 2.50) .0170	1.59 (0.93, 2.72) .0915
Q4 (0.946–2.12 g/L)	2.24 (1.51, 3.32) <.0001	2.09 (1.39, 3.13) .0004	1.68 (0.97, 2.93) .0663
*P* for trend	<.001	.0005	.2180

Model 1: no covariates were adjusted. Model 2: age and race were adjusted. Model 3: age, race, education level, income to poverty ratio, marital status, body mass index, weight, waist circumference, diabetes, hypertension, high cholesterol level, pelvic inflammatory disease, age at menarche, menstrual cycle regularity, female hormones, smoked at least 100 cigarettes, have 4/5 or more drinks every day were adjusted.

95% CI = 95% confidence interval, AGP = alpha-1-acid glycoprotein, OR = odds ratio.

### 3.3. Subgroup analysis

Table 3 presents subgroup analyses of the association between AGP and infertility, stratified by age, BMI, waist circumference, diabetes, hypertension, high cholesterol levels, menstrual cycle regularity, and use of female hormones. The results indicate that, across all stratified variables, the relationship between AGP and infertility did not differ significantly. Nonetheless, a noteworthy finding was the significant positive association between AGP levels and infertility observed in subjects under 35 years of age and in those with a normal BMI.

**Table 3:** Subgroup analysis for the between alpha-1-acid glycoprotein and infertility.

Subgroup	OR (95% CI)	*P* value	*P* for interaction
Age			.0903
<35	3.25 (1.04, 10.16)	.0429	
≥35	0.83 (0.28, 2.49)	.7374	
BMI			.1207
Normal weight	5.00 (1.14, 21.98)	.0333	
Overweight	1.23 (0.16, 9.26)	.8433	
Obesity	0.73 (0.25, 2.08)	.5516	
Waist circumference (cm)			.1388
<88	3.66 (0.85, 15.76)	.0810	
≥88	1.00 (0.41, 2.43)	.9929	
Diabetes			.1003
Yes	0.09 (0.00, 3.21)	.1891	
No	1.89 (0.86, 4.14)	.1143	
Hypertension			.0715
Yes	0.31 (0.05, 1.98)	.2155	
No	1.91 (0.83, 4.39)	.1268	
High cholesterol level			.5620
Yes	0.80 (0.09, 7.22)	.8439	
No	1.60 (0.70, 3.67)	.2639	
PID			.1357
Yes	0.03 (0.00, 6.94)	.2134	
No	1.66 (0.74, 3.69)	.2171	
Menstrual cycle regularity			.9010
Yes	1.58 (0.71, 3.55)	.2661	
No	1.89 (0.12, 28.67)	.6453	
Female hormones			.6095
Yes	0.31 (0.00, 177.49)	.7204	
No	1.63 (0.75, 3.54)	.2209	

In subgroup analyses stratified by age, BMI, waist circumference, diabetes, hypertension, high cholesterol level, pelvic inflammatory disease, menstrual cycle regularity, female hormones. The model adjusted for covariates such as age, race, education level, income to poverty ratio, marital status, body mass index, weight, waist circumference, diabetes, hypertension, high cholesterol level, pelvic inflammatory disease, age at menarche, menstrual cycle regularity, female hormones, smoked at least 100 cigarettes, have 4/5 or more drinks, but the model did not adjust for the stratification variables themselves.

BMI = body mass index, PID = pelvic inflammatory disease.

### 3.4. A nonlinear link between AGP and infertility

**Figure 2.**
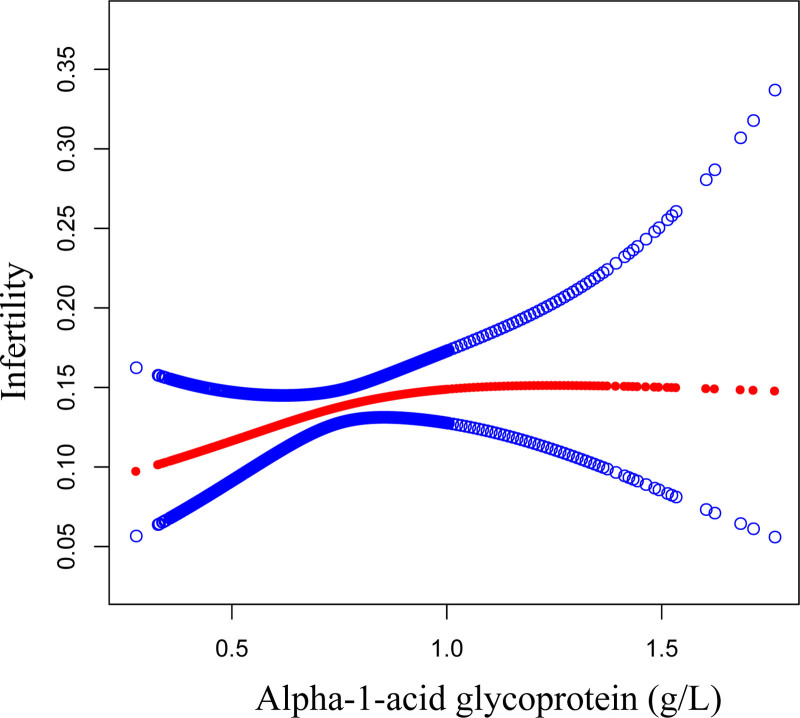
The nonlinear associations between alpha-1-acid glycoprotein and infertility. The solid red line represents the smooth curve fit between variables. Blue bands represent the 95% confidence interval from the fit.

**Figure 3.**
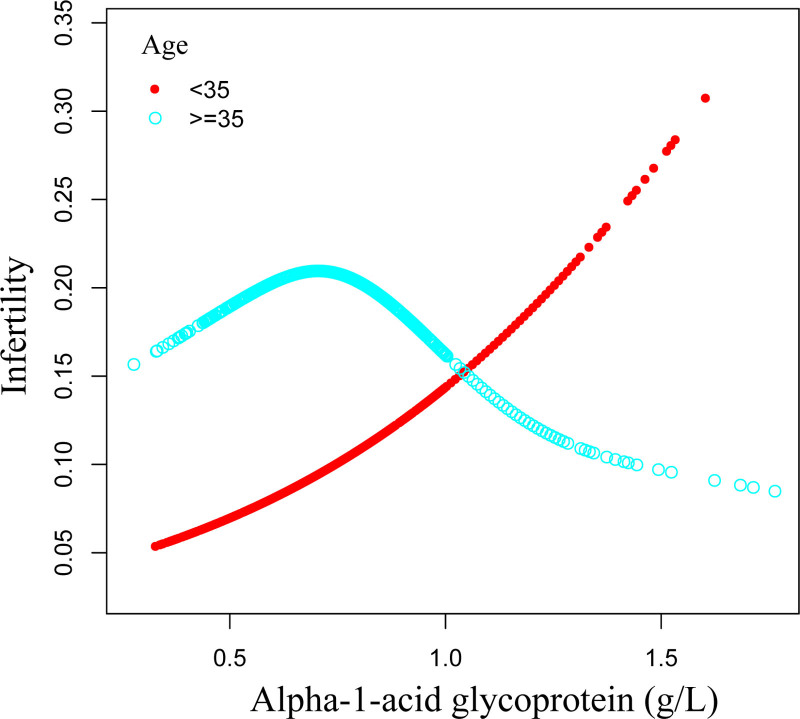
The nonlinear associations between alpha-1-acid glycoprotein and infertility stratified by age.

Smooth curve fitting methods were used to assess the nonlinear relationship between AGP and infertility, and the results showed a positive association (Fig. 2). Age-stratified analyses disclosed an inverse U-shaped association between AGP and infertility among women aged 35 years and older, with a critical threshold of 0.76 g/L (Table 4; Fig. 3). A non-significant inverse link was seen beyond this level (OR = 0.20, 95% CI: 0.04–1.02), but a substantial positive relationship was visible below it (OR = 16.20, 95% CI: 1.04–253.39) (Table 4).

**Table 4:** Threshold effect analysis of alpha-1-acid glycoprotein on infertility stratified by age.

Infertility	Adjusted OR (95% CI) *P* value
Stratified by age	
<35 years	
Inflection point (K)	1.19
AGP < 1.19	4.56 (1.22, 17.02) .0240
AGP > 1.19	0.31 (0.00, 133.02) .7031
Log likelihood ratio	.405
≥35 years	
Inflection point (K)	.76
AGP < 0.76	16.20 (1.04, 253.39) .0471
AGP > 0.76	0.20 (0.04, 1.02) .0529
Log likelihood ratio	.014

Age, race, education level, income to poverty ratio, marital status, body mass index, weight, waist circumference, diabetes, hypertension, high cholesterol level, pelvic inflammatory disease, age at menarche, menstrual cycle regularity, female hormones, smoked at least 100 cigarettes, have 4/5 or more drinks every day were adjusted.

95% CI = 95% confidence interval, AGP = alpha-1-acid glycoprotein, OR = odds ratio.

## 4. Discussion

This study examined a survey of 2229 women to assess the relationship between AGP and infertility. Our research revealed a strong association between the chance of infertility and those with higher AGP. Additionally, AGP and infertility had an inverted U-shaped connection in women over or equal to 35, with an inflection point of 0.76 g/L.

A substantial body of research indicates that inflammation plays a pivotal role in the etiology of infertility. From ovulation to egg implantation, fertilization, and pregnancy, inflammation affects almost every stage of the reproductive process; however, chronic inflammation reduces fertility.^[18]^ A study by Gloria revealed a significantly higher prevalence of infertility, 4-fold greater, among women aged 18 to 29 in the United States with a history of pelvic inflammatory disease.^[19]^ Research conducted by Yanfen Chen implicates lymphocytes in the pathogenesis of infertility, with elevated lymphocyte levels observed in individuals with infertility.^[20]^ A cohort study from Iran demonstrated that a pro-inflammatory diet is associated with increased systemic levels of chronic inflammation, which corresponds to a 76% higher risk of infertility.^[7]^ According to a prospective study by Jorge et al, ovulatory infertility was inversely correlated with an anti-inflammatory diet.^[21]^ The inflammatory milieu of the body can be improved by consuming less pro-inflammatory food and more anti-inflammatory foods. This can help avoid infertility and improve the health of the reproductive system.^[22]^ According to earlier research, infertility is typically linked to higher levels of several inflammatory variables, including C-reactive protein, interleukin-1 (IL-1), interleukin-6 (IL-6), and TNF-α.^[23,24]^ Our study, however, is the first attempt to assess the connection between AGP and infertility. During inflammatory events, AGP, an inflammatory marker primarily produced from the liver and peripheral tissues, can increase by up to 5 times.^[25]^ According to studies, women’s obesity and AGP are strongly related, and an accumulation of extra body fat may raise AGP.^[26,27]^ Additionally, obesity is one of the causes of infertility and might increase the risk of infertility.^[28,29]^ In a study by Medeiros, women with polycystic ovarian syndrome had greater levels of AGP, and AGP was linked to typical signs of inflammation such lymphocytes, neutrophils, and erythrocyte sedimentation rate.^[30]^ A recent cross-sectional case-control study examining inflammatory markers in women with PCOS revealed a significant difference in AGP levels between fertile and infertile groups.^[17]^ Specifically, the infertile cohort exhibited elevated AGP levels, which suggests that AGP may serve as a robust predictor of infertility within the PCOS population.^[17]^ Yao’s research highlights AGP as a significant biomarker for elucidating the underlying mechanisms associated with infertility in women afflicted with endometriosis.^[16]^

Our results are in line with other research showing a higher risk of infertility in those with higher AGP. Although the precise process by which AGP influences infertility is yet unknown, it may be connected to the following theories. Metamorphosis may be impacted by inflammation, which may result in epigenetic modifications in the endometrium that impact endometrial function and decrease endometrial tolerance.^[31]^ Ovarian senescence can also be influenced by inflammation, which can change the ovarian microenvironment and have an impact on fertilization and egg development.^[32,33]^ Between the ages of 35 and 40, age-related female infertility is increasingly clinically significant.^[34]^ Oocyte quality, oocyte quantity, and endometrial tolerance may all decline as women age.^[35]^ Endometrial function is altered by changes in gene expression in the endometrium starting at age 35.^[36]^

However, our research revealed an inverted U-shaped relationship between AGP and infertility as well as an AGP inflection point in individuals over or equivalent to 35. This differs from other studies and implies that there may be a more intricate relationship between inflammation and infertility in women over 35, which requires more research. Some such mechanisms are described below. The inflection point at 0.76 g/L in women ≥ 35 years represents a critical transition in inflammatory-fertility dynamics. Below this threshold, chronic low-grade inflammation (characterized by AGP elevation) accelerates ovarian aging through TNF-α-mediated mitochondrial dysfunction in oocytes, increasing reactive oxygen species generation and deoxyribonucleic acid fragmentation.^[37]^ Concurrently, AGP impairs endometrial receptivity by downregulating vascular endothelial growth factor and integrin expression, reducing endometrial thickness and vascularization.^[38]^ Above 0.76 g/L, systemic inflammation triggers compensatory immunosuppressive responses through regulatory T-cell expansion and IL-10 upregulation,^[39]^ while simultaneously activating stress-response pathways that enhance endometrial resilience through homeobox A10 overexpression.^[40]^ This biphasic pattern aligns with established principles of inflamm-aging, where moderate inflammation is detrimental but severe inflammation paradoxically induces protective mechanisms in reproductive tissues.^[41]^

Our study made use of a nationally representative sample of American women from a variety of ethnic backgrounds. We also conducted threshold effects analysis and several subgroup analyses to give a thorough evaluation of the relationship between AGP and infertility. Although this is one of our study’s strengths, there are still certain restrictions. Firstly, since this study is cross-sectional, causality cannot be inferred. Future longitudinal studies tracking AGP levels and infertility outcomes over time would provide stronger evidence of a causal relationship. Furthermore, infertility and covariates were self-reported, potentially introducing recall bias. While NHANES questionnaires are validated, underreporting of sensitive reproductive history cannot be ruled out. Third, although we adjusted for multiple covariates, residual confounding may still exist. Future studies should incorporate sensitivity analyses and additional covariate adjustments (such as genetic predisposition, environmental exposures, and lifestyle habits) to refine the association between AGP and infertility. Fourth, due to the limitations of the NHANES database, information on sterility was not accessible. Lastly, the modest sample size we used was reflective of the U.S. population and requires further research in larger samples and across various demographics.

## 5. Conclusions

Our study identified a positive association between AGP and infertility, with a statistically significant inflection point at 0.76 g/L among women aged ≥ 35 years. These findings may translate into a biomarker-guided clinical pathway: For women ≥ 35 years with AGP concentrations approaching 0.76 g/L, implementing anti-inflammatory dietary interventions (e.g., Mediterranean diet rich in omega-3 fatty acids) could reduce systemic inflammation and improve reproductive outcomes.^[21,42]^ For those with AGP > 0.76 g/L, adjunct metformin therapy may be considered given its established efficacy in reducing insulin resistance and improving ovulation rates.^[43]^ Nevertheless, large-scale prospective studies remain warranted to validate these observations and elucidate underlying mechanisms.

## Acknowledgments

We acknowledge the important contributions made by the study respondents, the NHANES database team, and our colleagues in the dermatology department.

## Author contributions

**Conceptualization:** Jia Lei.

**Data curation:** Jia Lei, Xiaohua Zhong.

**Formal analysis:** Jia Lei, Xiaohua Zhong.

**Methodology:** Jia Lei.

**Supervision:** Jia Lei, Xiaohua Zhong, Xiaohong Lin.

**Validation:** Meng Zhang.

**Visualization:** Jia Lei, Meng Zhang.

**Writing – original draft:** Jia Lei, Xiaohua Zhong.

**Writing – review & editing:** Jia Lei.
